# The oral microbiome of a family including Papillon-Lefèvre-syndrome patients and clinically healthy members

**DOI:** 10.1186/s12903-024-03856-z

**Published:** 2024-01-31

**Authors:** Péter Vályi, Roland Wirth, János Minárovits, Orsolya Strang, Gergely Maróti, Kornél L. Kovács

**Affiliations:** 1 Department of Oral Diagnostics, Faculty of Dentistry, Semmelweis University, Szentkirályi u 47, Budapest, H1085 Hungary; 2 Department of Biotechnology, University of Szeged, Közép fasor 52, Szeged, H6726 Hungary; 3 Department of Oral Biology and Experimental Dental Research, Faculty of Dentistry, University of Szeged, Tisza L. krt 64, Szeged, H6720 Hungary; 4 Institute of Biophysics, Biological Research Center, Temesvári krt 62, Szeged, H6726 Hungary; 5 Institute of Plant Biology, Biological Research Center, Temesvári krt 62, Szeged, H6726 Hungary

**Keywords:** Papillon-Lefèvre-syndrome, Periodontitis, *Aggregatibacter actinomycetemcomitans*, Oral metagenome, Periodontopathogens, Recessive mutation, Cathepsin C

## Abstract

**Aims:**

The oral microbiota composition of patients diagnosed with Papillon-Lefèvre-syndrome and treated for several years were compared to those existing in the oral cavity of the clinically healthy family members and a cohort of patients having various stages of chronic periodontitis.

**Materials and methods:**

A family with two sisters affected with severe periodontitis and with the typical skin symptoms of Papillon-Lefèvre-syndrome, and symptomless parents and third sibling were investigated. The Patients received periodontal treatment for several years and their oral microbiome was analysed by amplicon sequencing. Data were evaluated by microbial cluster analysis.

**Results:**

The microbiome of the patients with Papillon-Lefèvre-syndrome was predominated with *Aggregatibacter actinomycetemcomitans* and associated oral periodontopathogens. Although the clinically healthy family members showed no oral disorder, their microbiome resembled that of subjects having mild periodontitis.

**Conclusions:**

Predominance of *A. actinomycetemcomitans* in the subgingival microbiome of patients with Papillon-Lefèvre-syndrome suggests that specific treatment strategies directed against this pathobiont may improve the oral health status of the affected individuals.

**Trial registration:**

The study was conducted in accordance with the Declaration of Helsinki and the ethical permission has been issued by the Human Investigation Review Board of the University of Szeged, Albert Szent-Györgyi Clinical Centre (Permission No. 63/2017-SZTE). September 19, 2017. https://u-szeged.hu/klinikaikutatas/rkeb-altal-jovahagyott/rkeb-2017.

## Background

Papillon-Lefèvre syndrome (PLS) is a hereditary rare autosomal recessive condition affecting up to 5 people per million. An ectodermal dysplasia caused by loss of function mutations of the cathepsin C gene (*CTSC*), PLS is manifested as periodontitis and skin lesions [1]. It is remarkable that the PLS-associated and rapidly progressing severe periodontitis affects not only the primary dentition, but extends to the permanent dentition as well, leading to premature loss of both primary and permanent teeth. The other manifestation of PLS syndrome is a diffuse palmoplantar keratoderma characterized by hyperkeratosis and erythema on the palms, soles of the feet, elbows and knees [2–5]. Generally, the cutaneous lesions of PLS first appear between 6 months and 4 years of age, but in rare cases they may emerge in the first 3 months of life. Moreover, several cases of late-onset PLS have also been reported in the literature [6–8].

The etiology of PLS is not completely understood [9]. The primary cause of PLS has been identified as mutations in the *CTSC* gene, in the human chromosomal region 11q14.1-q14.3. CTSC is a lysosomal cysteine protease, also known as dipeptidyl peptidase 1 (DPPI). More than 100 mutations have been described including missense, nonsense or frameshift variants, which result in a reduced CTSC activity and coupled reduced host response against bacteria [10–14].

The *CTSC* gene is highly expressed in many tissues, such as the epithelial regions of the palms, soles and knees; keratinized oral gingival fibroblasts and osteoclasts [10, 15–17]. The majority of proteins that require processing by CTSC are part of the innate immune system [18]. Affected immune cells include natural killer cells, polymorphonuclear cells and T lymphocytes [19]. This may explain the multidimensional symptoms of PLS and may contribute to the development of malignant cutaneous neoplasms in certain PLS cases [20].

Polymorphonuclear neutrophils (PMNs) play a major role in the immune response against the key pathogens of various forms of periodontitis. Three strategies by which neutrophils serve as a first line of defence against invading pathogens are known as follows: i.) secretion of antimicrobial peptides (degranulation); ii.) engulfment of bacteria (phagocytosis); iii.) release of neutrophil extracellular traps (NETs) consisting of a nuclear DNA backbone associated with different antimicrobial peptides (AMPs) capable of capturing and killing pathogenic microorganisms [21].

Human neutrophils have an ambivalent role in the interactions with *Aggregatibacter Actinomicetemcomitans (Aa)*. Apparently, Aa. exploits neutrophils by inducing the exocytosis of azurophilic granules and the release of epinephrine, a catecholamine which activates QseBC bacterial two-component signalling system, facilitating thereby the survival and growth of *A. actinomicetemcomitans* under anaerobic conditions [22].

*A. actinomicetemcomitans* produces stabile biofilms that are resistant against removal by the flow of the saliva or the gingival crevicular fluid and highly resistant to antimicrobials as well, partly due the expression of the histone-like (H-NS) family of nucleoid-structuring proteins which facilitate the formation of multispecies biofilms [23]. In contrast LL-37, a peptid processed by protease cleavage of human CAP-18 (cathelicidine antimicrobial peptid 18) suppresses biofilm in vitro biofilm formation. LL-37 antimicrobal protein released from azurophilic granules of PMNs, is capable to bind and disrupt the membrane of various *A. actinomycetemcomitans* strains*.* Furthermore, LL-37 in sublethal dose also markedly suppressed in vitro biofilm formation and demonstrates opsonisation and agglutination enhancing thereby bacterial clearance by neutrophils and macrophages [24]. Thus evidence shows that LL-37 may act not only as a direct antimicrobial peptide, but also by its antibiofilm and immuno-modulatory properties.

CTSC is essential for the activation of three serine proteases (proteinase 3, neutrophil elastase and cathepsin G), which are components of the azurophilic granules of PMNs. Neutrophil azurophilic granules are partially responsible for the intracellular destruction of phagocytosed pathogens. Neutrophils are also a rich source of antimicrobial peptides (α-defensin 1-4 and cathelicidin). The human cathelicidin (hCAP18/LL37) gene (*CAMP, cathelicidin antimicrobial peptide*) encodes the peptide precursor human cationic antimicrobial protein (CAP), which is stored in the secondary granules of neutrophils as an inactive pro-form and gains antimicrobial activity via proteinase-3 cleavage, which generates peptide LL-37 - C-terminal part of the only human cathelicidin. Granular enzymes and peptides including neutrophil elastase (NE), myeloperoxidase (MPO), cathepsin G, leukocyte proteinase 3 (PR3), lactoferrin, gelatinase, lysozyme C, calprotectin, neutrophil defensins and cathelicidins are also involved in the antimicrobial activity of NETs [21].

The lack of functional CTSC in PLS patients leads to the absence of LL-37 in the gingival region [16, 25, 26]. PMNs are the main source of LL-37 in the healthy periodontium [26]. The levels of antimicrobial peptides in gingival crevicular fluids correlate with the prevalence and quantity of oral pathogens, predominantly *A. actinomycetemcomitans* in the subgingival plaque of PLS patients [24–26]. It is also noteworthy that bacterial proteases of other important periodontopathogens, such as *Porphyromonas gingivalis*, *Tannerella forsythia*, and *Treponema. denticola* might degrade hCAP18/LL-37 [26]. Severe congenital neutropenia (SCN, morbus Kostmann) is an autosomal recessive hereditary disease characterized by severe neutropenia resulting in LL-37 deficiency. Lack of LL-37 or very low levels of LL-37 in plasma, saliva and neutrophils of patients with Kostmann disease is associated with oral infections/inflammatory diseases like chronic periodontal disease. Although these patients are successfully treated by recombinant granulocyte-colony stimulating factor, which restores their levels of neutrophils, they have extremely low levels of both LL-37 and its precursor hCAP18 in plasma, saliva and neutrophils. For this reason, they present with symptoms of recurrent infections and periodontal disease [27]. A mutation causing a disruption of the neutrophil elastase enzyme was identified in the previously mentioned SCN patients. In another group of patients with Kostmann syndrome, we find a homozygous mutation of the *HAX1* gene that regulates neutrophil apoptosis, which is the most common SCN-related mutation in Turkey, and there is a variant in which neutropenia is not associated with the symptoms of Kostmann syndrome [28, 29]. Despite the fact that the level of antimicrobial proteins (HNP1-3, LL-37) in patients treated with G-CSF did not differ compared to the control group, these patients show more severe inflammatory signs, low bacterial diversity with high bacterial load associated with dysbiosis [29]. Severe congenital neutropenia treated by bone-marrow transplant may result, however, in functionally intact neutrophils and normal levels of LL-37 in plasma, saliva and neutrophils and, furthermore, periodontally healthy patients [30–32].

The evidence shows that LL-37 deficiency, either due to a deficiency of the enzyme required for the formation of the active form (PLS syndrome), or due to a low number or damaged neutrophils (Kostmann disease), causes severe, rapidly progressive, chronic periodontitis.

Numerous microbiological studies have identified the composition of the subgingival biofilms but there is limited information about the microbiological profiles of periodontal lesions in PLS. Species diversity was analysed with various microbiological approaches. Clonal analysis already indicated the importance of *A. actinomycetemcomitans* in the pathogenesis of PLS [25], which has been supported by increased levels of specific antibody against *A. actinomycetemcomitans* [2, 33]. Classical microbiological studies corroborated the outstanding predominance of *A. actinomycetemcomitans* in the subgingival plaques of PLS patients [26, 34–37]. Serine proteases, such as CTSC, are keystone enzymes in controlling the *A. actinomycetemcomitans* invasion. Low levels of LL-37 will render PMNs incapable of effectively neutralizing the leukotoxin produced by *A. actinomycetemcomitans*, further facilitating the aggression of *A. actinomycetemcomitans* in the subgingival plaque [16, 25, 38, 39]. Leukotoxin produced produced by *A. actinomicetemcomitans* causes activation of caspase-1 via NLRP-3 (NLR Family Pyrin Domain Containing 3) inflammasome which converts pro-IL-1β into active IL-1β and mediates its release from human macrophages, resulting in osteoclast differentiation and bone resorption. This mechanism correlated the onset and progression of periodontitis [40, 41].

Periodontitis is an inflammatory disease caused primarily by periodontopathogenic microbiota, whereas host defence mechanisms play an important role in its pathogenesis by modulating the local infection and eliciting the breakdown of periodontal tissues [42, 43].

Intrafamilial transmission of periodontopathogenic microorganisms is a well-known phenomenon [44–46]. The family involved in our investigation, was composed of both PLS patients carrying a seven-bases deletion in the *CTSC* gene and periodontally healthy members [47]. In this study we aimed the comprehensive characterization of the subgingival plaque microbiota of this family. We present the data on 3 siblings and 2 parents: two of the sisters developed typical symptoms of PLS, while the oldest sister and the parents were phenotypically healthy [47]. We compared the microbiota in samples from the PLS family with oral microbiota of adult chronic periodontitis patients as well as with data on samples from adolescents with and without gingivitis, presented earlier [48, 49]. Finally, we have also evaluated the clinical and microbial results of non-surgical periodontal treatment and adjunct antimicrobial therapy, applied in the case of the two sisters with PLS, designated as Patient-A and Patient-B respectively.

## Methods

### Study population

The study was conducted in accordance with the Declaration of Helsinki and the ethical permission has been issued by the Human Investigation Review Board of the University of Szeged, Albert Szent-Györgyi Clinical Centre (Permission No. 63/2017-SZTE). Informed written consent was gained from each subject at the commencement of the study and in case of participant under 16 years of age the informed written consent was obtained from their respective parents as well.

We have identified a Hungarian family with two sisters affected with severe periodontitis and with the typical skin symptoms of Papillon-Lefèvre syndrome, complicated with palmoplantar eruption. PLS was verified as a deletion in the *CTSC* gene, in the human chromosomal region 11q14.1-q14.3 [47]. The symptomless parents reported a third sibling of the affected daughters, who had no signs of PLS syndrome [47].

**Fig. 1:**
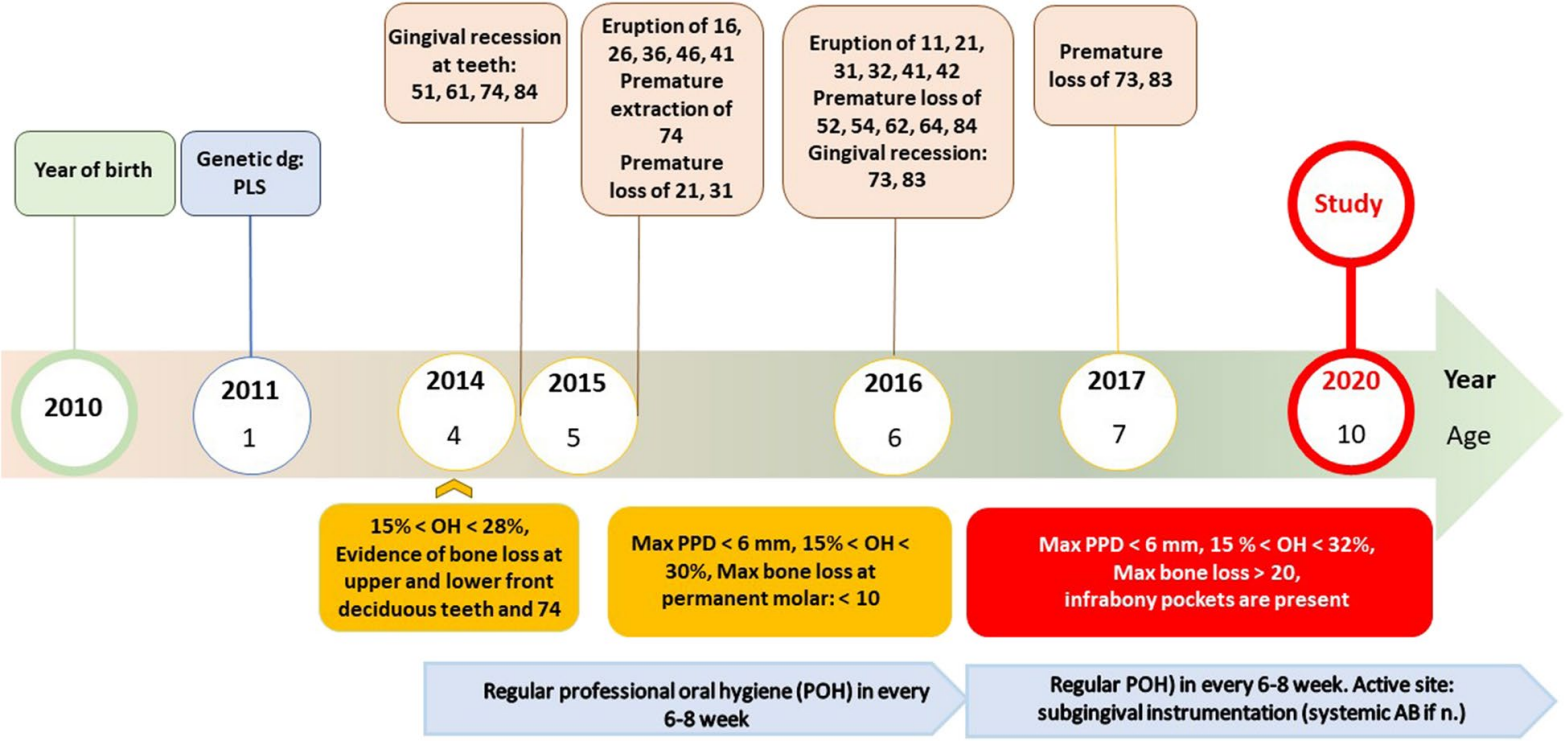
Timeline of Patient-A shows the most important events in disease course, periodontal parameters and treatment modalities of Patient-A

The youngest daughter suffering from PLS (Patient-A) was observed and examined first at the age of 2.5 years. Her regular treatment has started at age of 4,5 years, resulting in preservation of deciduous teeth until permanent teeth have erupted. By the age of 10 years, when this study was carried out, she had mixed dentition. Despite her compliance and good oral hygiene, active inflammation was seen at the permanent and adjacent deciduous teeth. Figure 1 shows the most important events in disease course, periodontal parameters and treatment modalities of Patient-A.

**Fig. 2:**
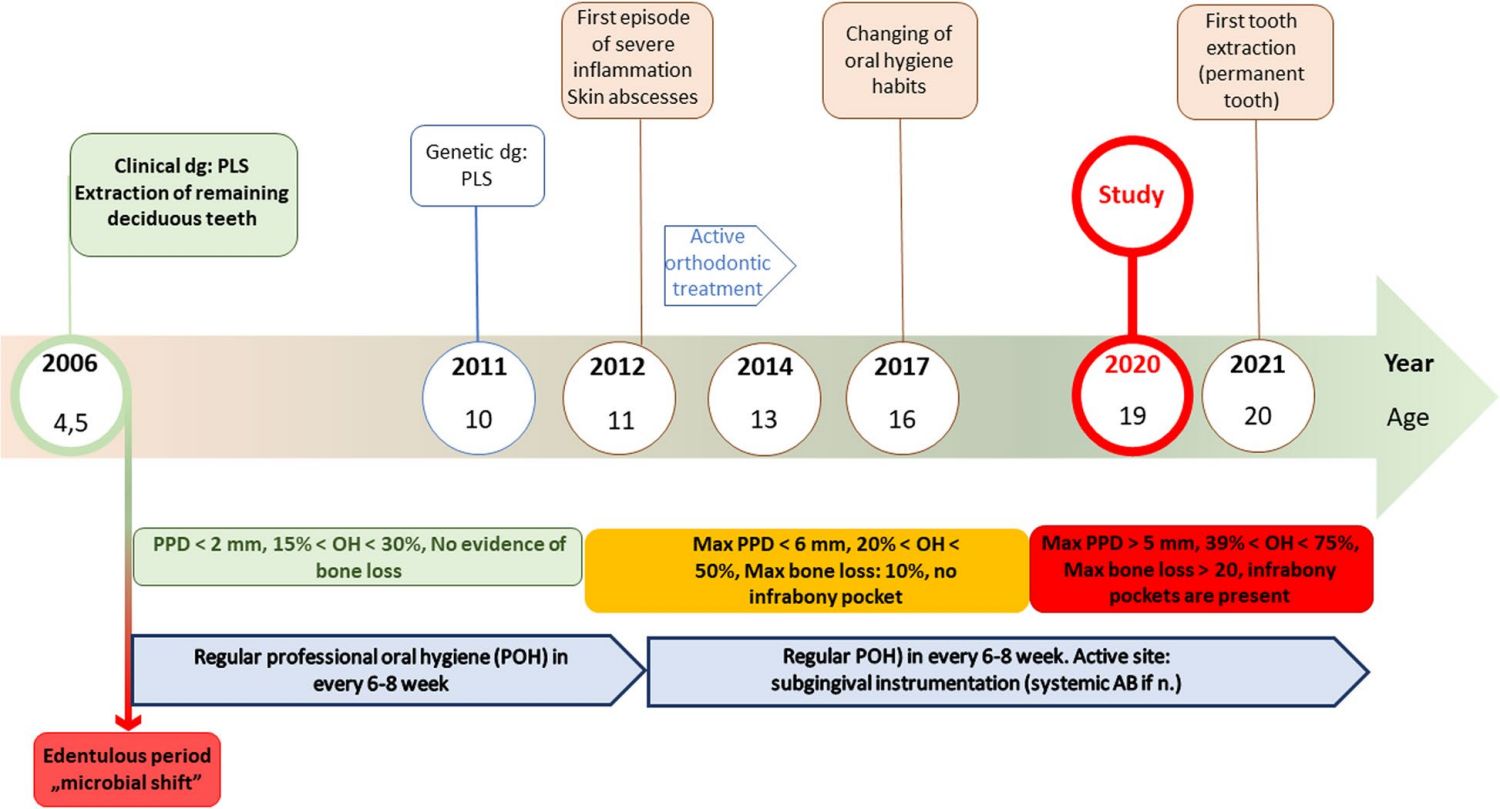
Timeline of Patient-B shows the most important events in disease course, periodontal parameters and treatment modalities of Patient-B

The older affected daughter (Patient-B) was 19 years-old at the beginning of the present study and has been treated regularly for 16 years. She has now moderate periodontal breakdown with active sites. She had lost deciduous teeth before the first molar erupted. Orthodontic treatment has solved several dental anomalies due to an early loss of deciduous teeth and partially the skeletal alterations. Successful maintenance of periodontal conditions has been reported to enable orthodontic treatment at the age between 11 and 13 within complex interdisciplinary therapy without further pronounced periodontal deterioration. Due to intensive, regular, professional oral hygiene care, the relatively healthy periodontal conditions were maintained for a prolonged period, until the patient retained her motivation and paid sufficient effort on oral hygiene at age of 16 [50]. One year after study finished, she lost her first permanent tooth. A timeline (Fig. 2) indicates the most important events in disease course, periodontal parameters and treatment modalities of Patient-B.

PLS patients received regular supportive periodontal therapy in 6–8-week intervals. Active inflammatory episodes have been treated by non-surgical methods.

Examination of the parents and the oldest sister revealed that they were periodontally healthy according to criteria of the new classification of periodontal diseases and conditions [51]. The oldest sister (Subject-C) is 22 years-old with intact dentition, without any signs of gingival inflammation and periodontal destruction supported by excellent oral hygiene (plaque index PI< 10%). The symptomless father (46 years-old, Subject-D) and mother (44 years-old, Subject-E) are in the middle-ages with good oral hygiene (PI< 20%). Neither Subject-D nor Subject-E displayed any clinical or radiological signs of periodontal destruction or bleeding on probing.

### Periodontal examination

The periodontal parameters of PLS patients were registered at 3-6-month intervals during the years. The following clinical parameters were measured at six sites per tooth by previously calibrated examiners: probing pocket depth (PPD), clinical attachment level (CAL), bleeding on probing (BOP), and plaque control record (PCR) [52] using a CP 15 periodontal probe [48]. Radiographic orthopantomogram (OPG) examinations were carried out yearly, whereas periapical radiographs of problem areas were taken when the situation required. In addition, comprehensive periodontal examinations were carried out and OPGs were taken to assess the periodontal condition of healthy family members as well.

### Microbiological sampling

Microbiological samples were collected from 8 affected periodontal pockets (PPD > 4 mm, BOP +) of each PLS patient, and 4 healthy sites (PPD < 3 mm, BOP -, no evidence of carious lesion) were sampled in case of the clinically healthy family members. When selecting the test sites, we took care take samples from all quadrants and all types of teeth for representative. The supragingival plaque was removed with sterile cotton pellets and the sampling site was isolated with cotton rolls prior to sampling. A sterile paper point was inserted to the bottom of the periodontal pocket and kept there for 30 s. In the case of unaffected family members, the gingival sulcus was sampled in a similar manner. The paper points were placed into separate empty sterile plastic tubes. The sampling procedure has been repeated in case of PLS patients 3 months after at the next recall appointment.

### Periodontal treatment

PLS patients needed periodontal treatment of acute inflammation at the start of study. After microbiological sampling, the PLS patients were treated by mechanical, non-surgical treatment and have received systemic antibiotics at same time. Antibiotics were not administered for at least 6 months prior to the therapy and the same active agent was not used for at least a year. Full-mouth supra- and subgingival debridement was carried out using an ultrasonic device and single root planning of diseased sites under local anesthesia. Additionally, the older sister (Patient-B) was treated with a single regimen of doxycyclin, 100 mg two times daily for 10 days for the primary purpose of preventing further destruction of supporting tissue, when her plaque index reached 33% (Table 1), while the younger sister (Patient-A) received 250 mg metronidazole three times daily for 7 days. Both patients received oral hygiene instructions and reinforcement of motivation. The normal recall interval has extended due to COVID lockdown.

**Table 1:** Periodontal parameters of the affected PLS sisters before and after acute episode of inflammation

		Before treatment	After treatment	*p* value
Subject A	PPD	8,25 ± 0,71 mm	6,25 ± 1,28 mm	*p = 0,01*
	BOP	31,30%	28,60%	
	PCR	18,20%	24,50%	
Subject B	PPD	7,13 ± 2,36 mm	4,88 ± 1,73 mm	*p* = 0,14
	BOP	27,30%	45,60%	
	PCR	33,90%	44,50%	

### DNA isolation

Subgingival plaque samples, taken by sterile paper points, were resuspended in 500 μl TE buffer (10 mM Tris-HCl, 1 mM Na-EDTA, pH 8) and were pelleted at 13,000 rpm for 5 min. DNA extractions were carried out by using the Macherey-Nagel (Düren, Germany) NucleoSpin Soil DNA kit (Macherey-Nagel:740780.250). Sample preparation and quality estimation were performed according to [48].

### Next-generation sequencing

The amplification, purification, and sequencing of the prokaryotic hypervariable V3-V4 region of 16S rRNA gene were performed as described in “Preparing 16S Ribosomal RNA Gene Amplicons for the Illumina MiSeq System” standard protocol provided by the supplier (Illumina). Prokaryotic 16S rRNA gene amplification, purification, and sequencing were done as described in the standard protocol of the supplier (Illumina, San Diego, CA, USA). Briefly, the hypervariable V3-V4 region of the 16S rRNA gene was PCR-amplified, and DNA sequencing was carried out on an Illumina MiSeq machine using V2 sequencing chemistry (MiSeqReagent Kit v2) (500 cycles). Detailed description of the applied method has been published previously [48].

### Amplicon sequence analysis

Amplicon sequencing data were handled employing an in house-developed bioinformatics pipeline, which comprises five modules [48] as follows. 1) Sequencing preparation: join paired end fastq reads ($cat). 2) Trimming: raw sequences were trimmed by Trimmomatic (v.0.36.5:slidingwindow: 4:20, minlen: 200, leading: 3, trailing: 3) and checked with FastQC (v.0.11.8) [53]. 3) Taxonomic annotation: amplicon sequences were annotated with Kraken2(v.2.0.8) using the NCBI RefSeq (genome) database. 4) Filtration and normalization: Kraken feature table outputs were filtered by Kraken2 (−confidence 0.95) [54]. Copy number normalization was done through the rrnDB (v.5.6) [55] database. MetagenomeSeq (v.1.16.0) was used to create normalized and scaled output of microbial abundances (−rel 0.1,–scale 1000) [56]. 5) Statistics and visualization: Megan6 (v.6.18.1) was used to export data for statistical calculation [57].

### Statistical analyses

For statistical analysis and visualization STAMP, R studio microViz and microeco packages were used. Alpha diversity was calculated with Shannon statistical method (R microViz) [58], and Bray-Curtis distance applied for beta diversity (R microeco) [59]. Significantly different taxa were identified by STAMP (Statistical Analysis of Metagenomic Profiles) [two-sided t-test with 0.95 confidence intervals (*p* ≤ 0.05)] [60] and Benjamini–Hochberg false discovery rate (FDR) was used in order to filter out false-positive significant differences. Unweighted pair group method with arithmetic mean (UPGMA) with Bray–Curtis method was employed to cluster the samples and visualized by interactive Tree of Life (iTOLv.5.3) online platform [61].

Statistical analysis of clinical parameters was carried out by Statistica (version 13.5.0.17, TIBCO Software Inc., Palo Alto, California, USA). Non-parametric tests (Wilcoxon-test in group and Kolgomorov-Smirnov test between group) were performed.

## Results

### Clinical parameters

The periodontal parameters are presented in Table 1. The average of periodontal pocket depth (PPD) was similar both before and after treatment in the two affected patients. Significant improvement could be found in case of the younger sister (Patient-A), probably due to the non-surgical periodontal therapy. The plaque control record (PCR) values worsened in both cases, although the bleeding on probing (BOP) improved in Patient-A 3 months after the treatment. Although there was an increase in BOP and PCR, Patient B also showed a decrease in PPD.

### Diversity of the microbiomes

**Fig. 3:**
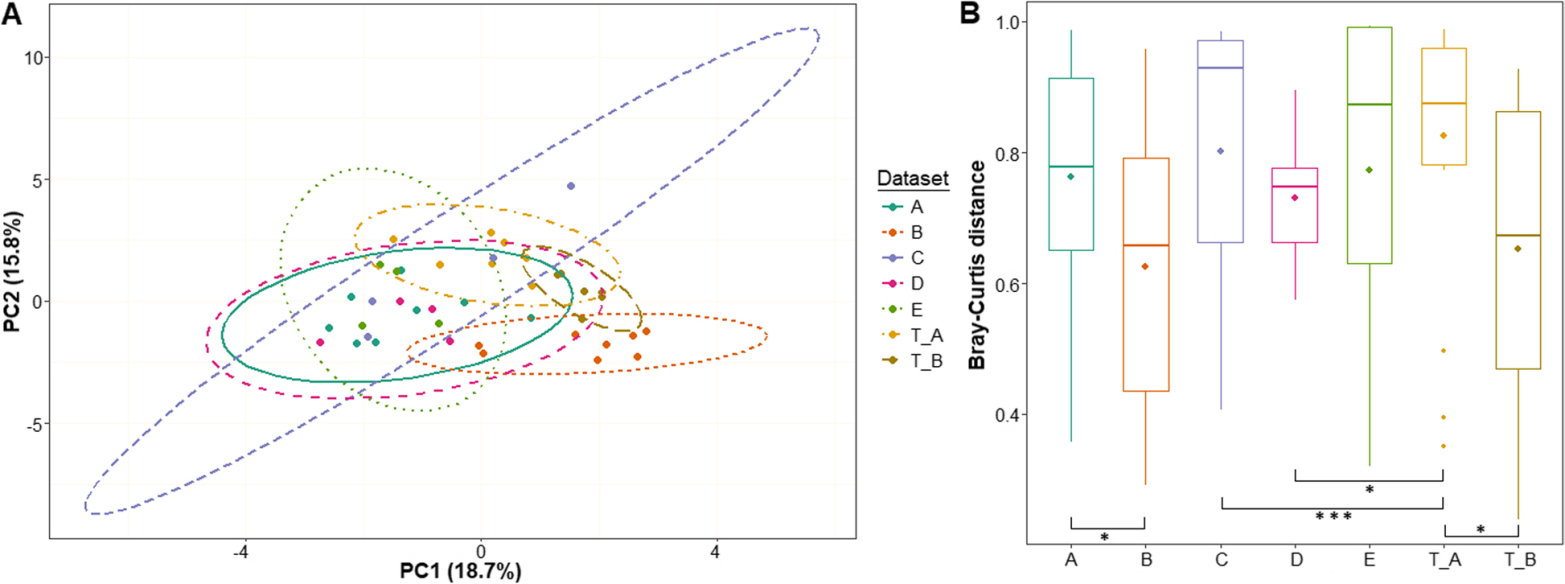
Principal Coordinate Analysis (PCoA) and beta diversity. **a** PCoA. PC1 and PC2 dimensions represents 15.8 and 18.7% of the microbiome variation between data. According the PCoA, the sample groups are not significantly different (proven by PERMANOVA test, data not shown). Nevertheless, significant distinction is seen when the PLS Patients-A and -B (A and B, solid lines) are compared to the clinically healthy family member Subjects C-D-E (C, D, E, dashed lines) before and after treatment (T_A and T_B, dotted lines). **b**. Beta diversity. There were significant differences between the sample groups. Cutpoints are 0-0.001, 0-0.05, 0-1 and the corresponding symbols are “***”, “*”, “ns” (not significant)

The PCoA and beta-diversity analysis of the data sets of the 5 family members indicate a high degree of dissimilarity among the subgingival microbiomes (Fig. 3). Remarkably there are “outlier” microbiomes in each sample set indicating a substantial heterogeneity of the bacterial communities within each subjects’ oral cavity regardless of the state of disease (Fig. 3b) Nevertheless, the microbiomes of the clinically healthy Subjects-C-D-E form a group, distinct from that of Patient-A and -B. As far as dissimilarity index (Bray-Curtis distance) can be decisive, treatment altered the microbiome of Patient-A, but did not affect substantially the microbiome of Patient-B (Fig. 3b).

### The subgingival microbiomes of the PLS patients and apparently unaffected family members

**Fig. 4:**
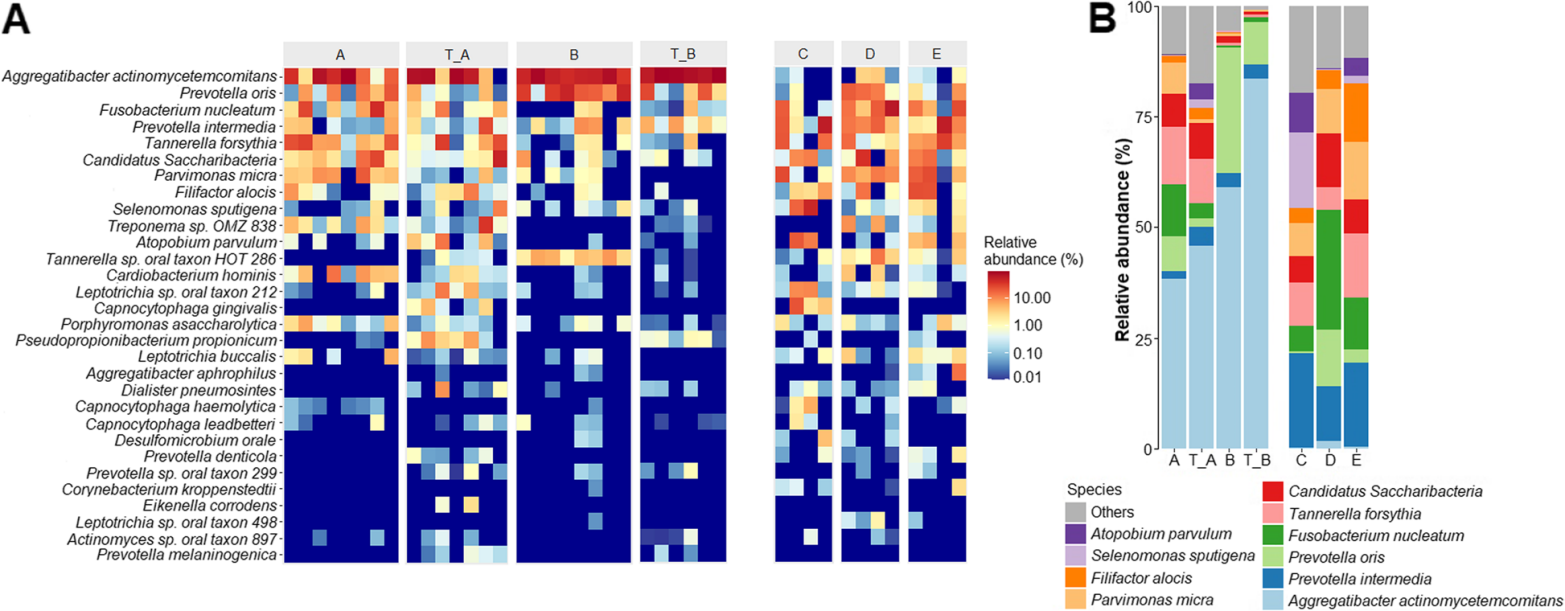
Abundance of bacterial species. **a** Heat map distribution of the most abundant 30 bacterial species. Subgingival bacteria of PLS patients before (A and B) and after (T_A and T_B) treatment as well as bacterial taxa of the clinically healthy family members (C, D, E) are shown in the columns from left to right. **b** Relative abundances of the top 10 bacterial strains. Subgingival bacteria of PLS patients before (A and B) and after (T_A and T_B) treatment as well as and bacterial taxa of the clinically healthy family members (C, D, E) are shown

**Fig. 5:**
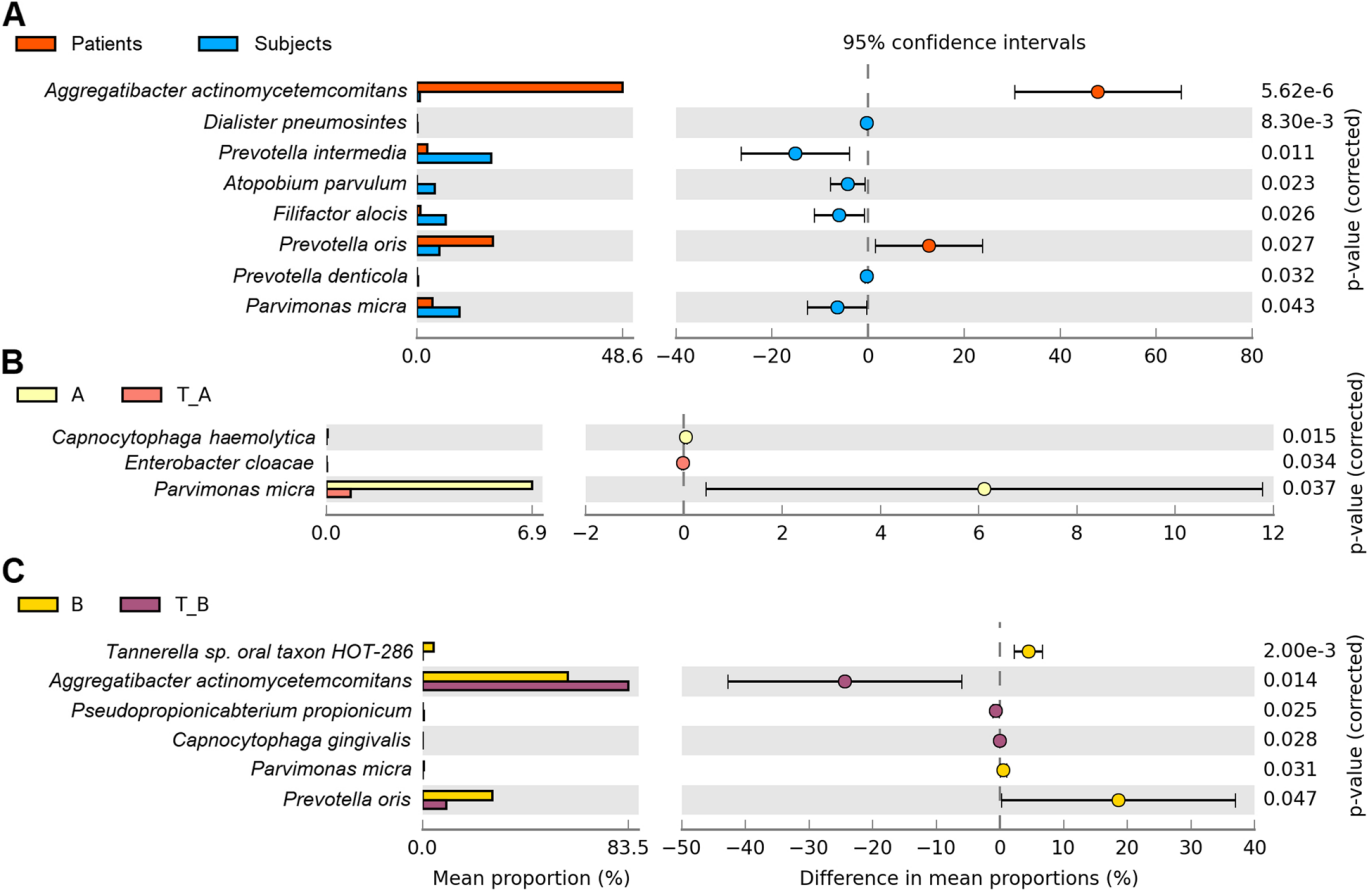
Significant differences between subgingival microbiota. **a**, PLS Patients versus clinically healthy family members. Note the relative abundance increase of A. aggregatibacter and *P. oris* (indicated in red) and the diverse oral pathogens detected in the oral cavity of the clinically healthy family members (blue columns) **b**) and **c**,) Significant differences prior and after the periodontal therapy in Patients-A and B, respectively

All samples from each PLS patient before and after treatment and the samples from the clinically healthy family members have been compared (Fig. 4). The heat maps of the most abundant 30 identified taxa (Fig. 4a) illustrate both the diversity of the microbiomes in the oral cavity of the individual subjects and the prominent differences between the two PLS patients and their clinically healthy family members. The massive difference in the relative abundance of *A. actinomycetemcomitans* between the PLS (Patients A and B) and “healthy” (Subjects C-D-E) groups is apparent in the individual samples at first glance (Fig. 4a). We noted that this *A. actinomycetemcomitans* predominance did not diminish upon treatment in either patient. Hence *A. actinomycetemcomitans* cannot be considered as the diagnostic marker strain of PLS. This is in contrast with the clinical observation of an almost complete permanent denture in Patient-B. At any rate, *A. actinomycetemcomitans* is predominant in the diseased microbiome while it is a marginal species in the microbiome of the clinically healthy family members. Unlike *A. actinomycetemcomitans*, *Prevotella oris*, the second most abundant oral pathogen in the periodontal pockets of the PLS patients seemed to respond positively to the treatment in case of Patient-B. *P. oris* was not predominant member of the microbiome in the clinically healthy sister (Subject-C) and mother (Subject-E) (Fig. 4b). Similarly, *Parvimonas micra* responded positively to the tetracycline treatment in Patient-A (Fig. 5b). Interesting distinctions were observed in the relative abundances of oral pathogens *Fusobacterium nucleatum* and *T. forsythia* between the two PLS patients. Both pathogens were present at low relative abundance in Patient-B, which did not change substantially upon treatment. The differences between the PLS versus healthy microbiota are clearly distinguishable when the average abundances of the sample groups, i.e., diseased, treated patient, clinically healthy, are appraised (Fig. 4b). The relative abundance of the genera *Prevotella*, *Fusobacteria*, *Tannerella* as well as the *Candidatus Saccharibacteria* behave similarly, i.e. their relative abundance was reduced in the PLS patients relative to the clinically healthy family members and this composition did not change considerably upon treatment.

A pairwise comparison of the significant differences among the diseased (Patients), treated patients and clinically healthy (Subjects) groups (Fig. 5) corroborated the findings summarized in Fig. 4. The largest difference between the PLS positive (Patients A and B) and clinically healthy family members (Subjects-C-D-E) was in *A. actinomycetemcomitans* (Fig. 5a). Notably, there was a fairly pronounced distinction between Patient-B and Subject C-D-E in the abundance of the genus *Prevotella*, but the relative representations of the individual strains diverged. The prevalence of *A. actinomycetemcomitans* in the diseased oral cavity did not diminish after the dental treatment regime relative to the clinically healthy family members (Fig. 5). The large predominance of *A. actinomycetemcomitans* in the PLS patients did not change significantly in Patient-A upon the non-surgical dental treatment and metronidazole administration as no significant difference in its abundance could be detected between the “before” and “after” treatment (Fig. 5b). In contrast, the predominance of *A. actinomycetemcomitans* increased in Patient-B (Fig. 5c). *P. oris* and *P. micra* persisted as leading oral pathogens but responding positively to the treatments in Patients-A and -B, respectively (Fig. 5b and c).

### The microbiological clusters

**Fig. 6:**
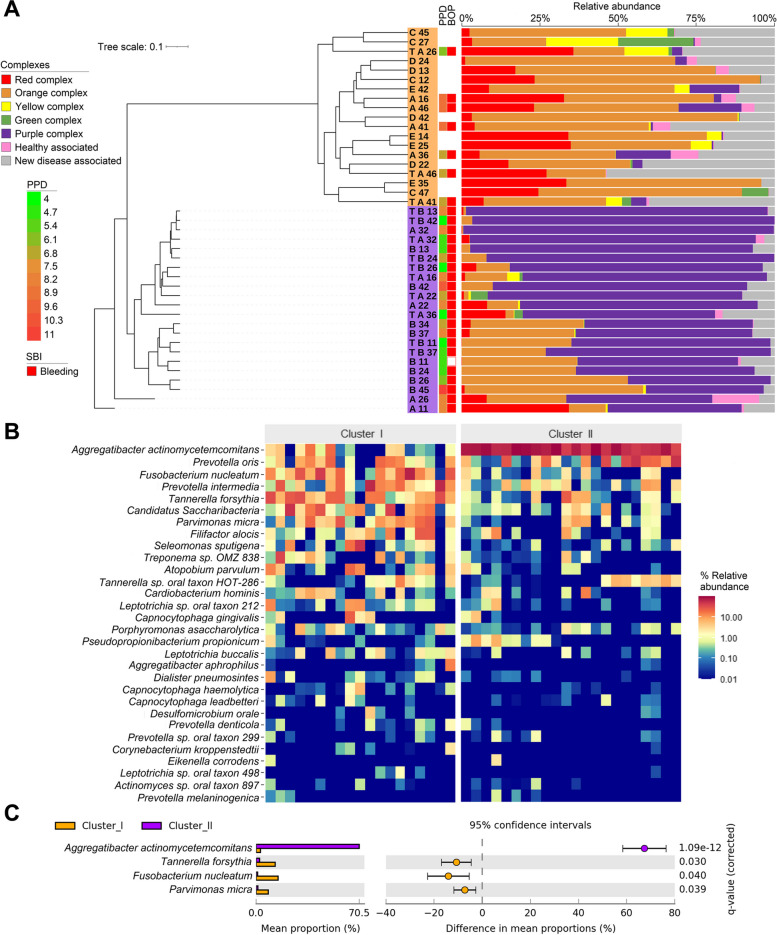
Clustering of microbiomes. **a**. UPGMA (unweighted pair group method with arithmetic mean) tree reveals two microbial clusters. The column leftmost from the tree indicates the positions of the sampled teeth in orange and purple background columns. Next the Periodontal pocket depth (PPD) and Bleeding on probing (BOP) relative values are indicated. The colors in the horizontal bars mark the corresponding elements of the Socransky pyramid. The lengths of the columns are proportional to the relative abundance of the given Socransky group. **b**. Heat map distribution of the most abundant 30 species in the microbial clusters. **c**. Significantly different species between the two microbiological clusters. Please nore that these significantly different microbes are not the same as in Fig. 3

Irrespective of the family members and treatment history, the microbiomes of the samples can be arranged into two clusters (Fig. 6) following the Socransky colour coding of oral microbes [62–64] as modified in Wirth et al., 2021 [48]. The microbiomes of the total 41 sequenced samples readily split into two clusters (Fig. 6a), which will be referred to in the subsequent analysis as the orange-dominated Cluster-I and purple-dominated Cluster-II, respectively. The colours indicate the predominant colour coded grouping in the Socransky pyramid. Dissecting the two clusters revealed that all microbiomes falling into the purple Cluster-II belonged to the PLS Patient group. Only 7 out of the total 28 microbiomes from PLS Patient group (A16, A36, A41, A46, TA26, TA41, TA46) mapped in the orange Cluster-I. It is noteworthy that all of these 7 microbiomes belonged to Patient-A, who developed the PLS clinical symptoms strongly and lost all deciduous teeth at the age of 4.5 years. *A. actinomycetemcomitans* was by far the most abundant oral pathogen in the purple Cluster-II although the purple complex is usually considered less pathogenic than the members of red and orange complexes [62, 65, 66]. It is noteworthy that the Cluster-1 samples, comprised mostly of the PLS Patient-A and Patient-B, contain microbial taxa belonging in the red and orange Socransky complexes. In contrast, the samples from the clinically healthy family members (Subjects-C-D-E) group in the Cluster-I microbiomes. The clinical symptoms, i.e. probing pocket depth (PPD) and particularly bleeding on probing (BOP) were more severe in the samples belonging in Cluster-I than in Cluster-II (Fig. 6a). It is noteworthy, that the damaging clinical symptoms are associated with the apparently less pathogenic microbiomes predominated by *A. actinomycetemcomitans*, i.e., Cluster-II. Dental treatment did not eradicate the *A. actinomycetemcomitans* prevalence although most of the permanent teeth of Patient-B had been preserved as long as the patient maintained oral hygiene properly.

The heat map of the relative distributions of taxa (Fig. 6b) clearly depicts the main differences between the two Clusters. Cluster-I was more diverse and the prevalence of various oral pathogens, such as *P. oris*, *F. nucleatum*, *Prevotella intermedia*, *T. forsythia*, *C. Sacharobacteria*, *P. micra* and *Filifactor alocis* was apparent. Cluster-II was overwhelmingly predominated by *A. actinomycetemcomitans*. In addition to the diversity of Cluster-I, this microbial community is dissimilar from the characteristic “healthy” oral microbiome.

### The correlation/co-occurrence analysis

The clinical symptoms, i.e., probing pocket depth (PPD) and particularly bleeding on probing (BOP), were more severe in the samples belonging in Cluster-II than in Cluster-I.

**Fig. 7:**
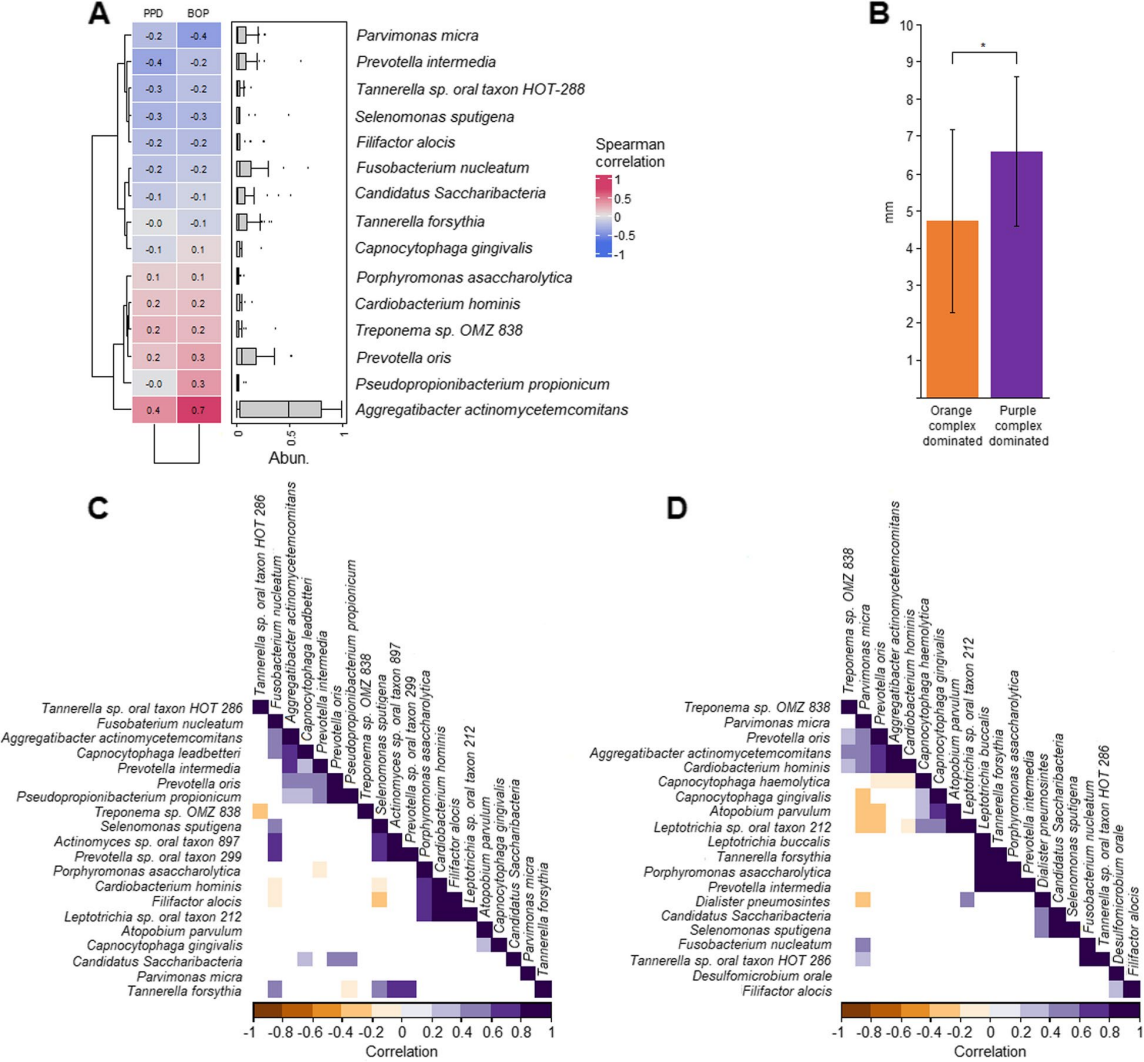
Correlation and co-occurrence analysis results. **a** and **b**: Correlation between clinical parameters and microbiota. PPD: probing pocket depth (PPD); BOP: bleeding on probing. **c** and **d**: Co-occurrence analysis in microbial Cluster-I and -II

A correlation/co-occurrence analysis was therefore carried out (Fig. 7). The Spearman correlation analysis suggests that the two most important clinical parameters, PPD and BOP show strong correlation with *A. actinomycetemcomitans* only. Weak additional correlation of these parameters with *P. oris*, *Treponema* sp., *C. Saccharibacteria*, and perhaps *Cardiobacterium hominis*, could be detected (Fig. 6c).

A strong and significant correlation was detected between the clinical parameters and microbiota composition in a Spearmen analysis (Fig. 7a and b). Cluster-I and Cluster-II also differed in their partnership associations. In the purple Socransky complex predominated Cluster-II (Fig. 7c and d) *A. actinomycetemcomitans* teamed up with a number of periodontopathogens, e.g., *F. nucleatum*, members of the *genus Prevotella*, *T. forsythia*, *C. hominis*, *Leptotrichia* sp., *Pseudopropionibacterium propionicum*, *F. alocis*. Taken together, pathogenic oral microbial communities prevailed in both PLS-associated Clusters, albeit the ostensible differences. Cluster-I microbiomes signal the apparently healthy family members, whereas the microbial community of Cluster-II samples should serve as an indicator of the microbiological conditions of PLS patients (Fig. 7c and d).

### The PLS family microbiomes are different from the oral inflammatory microbiomes

**Fig. 8:**
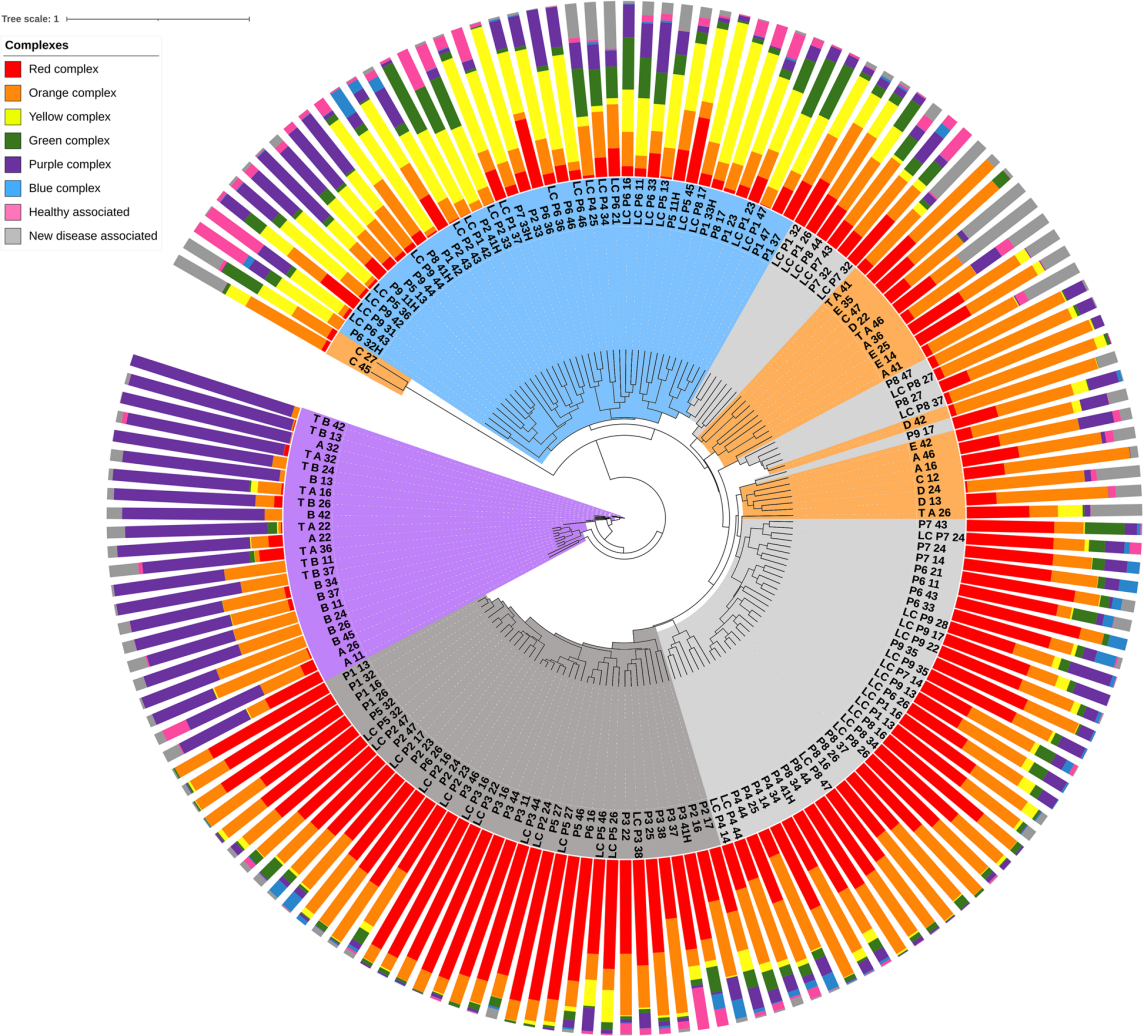
Comparison of PLS family microbiota and subgingival bacteria of periodontitis patients. The positions of subgingival microbiomes from PLS Patients-A/B and their healthy family members (Subjects-C-D-E) are shown, relative to a population of chronic periodontitis patients) [37, 38]. Numbers marking the anonymous individual patients and sampled tooth positions are highlighted in dark grey (=severe periodontitis), light grey (=periodontitis), and light blue (=healthy) backgrounds, according to their position on the major branches of the UPGMA tree (innermost ring #1). The microbiomes (orange) of the clinically healthy PLS family members map in the light grey area of Ring #2. The microbiomes of PLS Patients-A/B form a distinct branch (purple in Ring #1. Composition of microbial communities according to the Socransky complexes are indicated by the columns (outmost ring #3)

Next, we compared the microbiomes of the PLS family members, including the PLS Patients-A, Patient-B and the clinically healthy family members (Subjects-C-D-E) with the microbiota of patients affected with chronic periodontitis and gingivitis [48, 49]. It should be noted that sampling, sample treatments, sequencing and sequence data evaluation had been done essentially in the same environment by the same experts and using the same methodology in the three sets of experiments. Therefore, the data obtained are comparable. The PLS metagenomes were mapped on a fairly large compilation of adult, chronic periodontitis microbiome database (Fig. 8). The taxonomic tree (centre of Fig. 8) already indicates a pronounced separation of the microbiomes of the PLS Patients-A/B from the rest of the samples (innermost ring, purple background, Cluster-II in Fig. 8). The PLS Patients’ microbiome are disconnected from any of the periodontal disease microbiomes (light and dark grey background, innermost ring) or the healthy controls (blue background, innermost ring). Predominated by the members of the Socransky’s purple complex microbes, namely *A. actinomycetemcomitans*, the microbiome of PLS Patients-A/B represent a group, which seems to be completely dissimilar to any other, “classical” periodontitis microbiome.

Interestingly, the microbiomes of the apparently healthy family members (Subjects-C-D-E, highlighted by the orange background in Fig. 8) do not map to the “periodontally healthy” (blue background in the innermost ring of Fig. 8) group. Instead, they fall within the “moderately diseased periodontitis” cluster (light grey background in the innermost ring in Fig. 8). This corroborates our earlier findings indicating that the apparently symptomless family members do not possess a healthy microbiome, just a diseased one without clinical signs of the disease. This indicates that even a heterozygous recessive gene mutation can bring about considerable alterations at the microbiome level, which has not been recognized before.

**Fig. 9:**
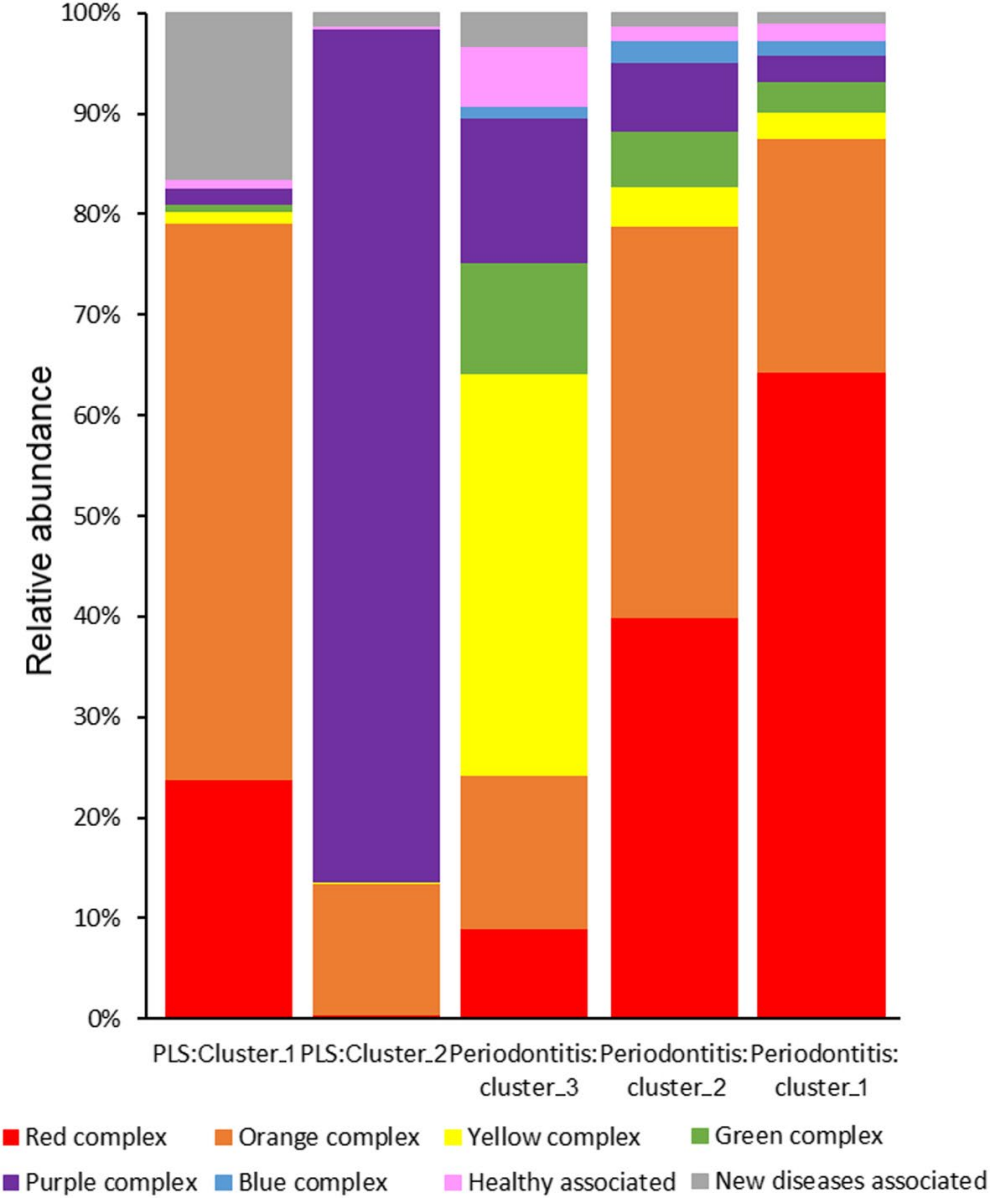
The distribution of relative abundances of the various Socransky groups in the microbial clusters of the PLS Subjects-C-D-E (Cluster-I) and PLS Patients-A/B (Cluster-II) with the chronic periodontitis cluster microbiomes

The comparison of the average predominance of the Socransky complexes among the various orally affected patient groups suggest the same general principle to be valid in a larger cohort as well. Figure 9 compares the major microbial complexes investigated in this study. At first glance it is apparent, that the PLS Cluster-II, i.e., diseased PLS Patient-A and Patient-B, the predominance of the purple complex exceeds any other oral microbial community composition studied so far. The PLS “apparently healthy” family members (Subjects C-D-E) harbour a microbial community resembling that of the periodontitis Cluster-2 [37], but any comparison between these study groups delivers no additional novel findings.

### Microbial effect of periodontal treatment

The relative abundance of *A. actinomycetemcomitans* is apparently crucial in the subgingival microbiome of PLS patients. Three months after non-surgical periodontal therapy with two distinct adjunctive antibiotics regimen resulted in increased proportion of *A. actinomycetemcomitans* in the subgingival microbiome. In Patient-A, who received metronidazole, the proportion of *T. forsythia*, *P. micra*, *F. nucleatum* and *P. oris* decreased while the occurrence of other periodontopathogenic taxa increased. The microflora of Patient-B became simpler, i.e., more than 60% consisted of *A. actinomycetemcomitans.* The non-pathogenic taxa were minimized and the level of *P. oris* dramatically decreased. Other significant alterations were not judged relevant in Patient-B.

## Discussion

It was suggested that immune defects affecting neutrophils play an important role in the pathology of periodontal disease [67]. Our study indicates that – possibly on the basis of, or in addition to such dysregulated immune alterations - in PLS patients one may observe an altered composition and a decline in the diversity of the subgingival microflora compared to healthy family members. In PLS patients we detected a higher relative abundance of several periodontal pathogens, especially *A. actinomycetemcomitans,* which increased after mechanical and antimicrobial therapy. Our results demonstrated that antibiotics could enhance the beneficial clinical effects of mechanical periodontal therapy, and improve the clinical outcome, only in patients with good oral hygiene compliance. We observed that although the healthy subjects had quite good clinical parameters, composition of subgingival microflora consisted predominantly of periodontal pathogens. Thus, they fell within the “moderately diseased periodontitis” cluster.

The oral phenotype of the two PLS sisters in this study partly followed the syndrome profile but also showed considerable differences between the affected individuals. Although the oral habits changed in case of the older affected (Patient-B), the relatively healthy periodontal conditions were maintained for a prolonged period thanks to the premature loss of deciduous teeth and a consequent “microbial shift”, in accordance with the conclusions of several studies [68–71]. Early loss of primary teeth influences the development of jaw, the position of the lip as well as the position of the permanent teeth underlying the need for orthodontic treatment [72].

In our case, the early regular periodontal treatment of younger affected sister (Patient-A) resulted in the maintenance of deciduous teeth prior to the eruption of permanent teeth. Deep periodontal spaces developed during eruption at the adjacent primary teeth after exfoliation of secondary teeth, providing opportunity to infection. Despite of meticulous individual and professional oral hygiene and periodontal treatment, the periodontal destruction of the younger affected patient (Patient-A) became more severe relative to her sister (Patient-B).

In previous studies, various methods were applied to analyse distinct microbiomes in PLS patients. Albandar et al. presented a subgingival composition of microflora in PLS patients, based on clonal and microarray analysis of 16S rRNA genes [34], whereas Lettieri described the salivary microbiome of PLS patients by next generation sequencing [33]. In line with these early studies, we also noted a higher relative abundance of *A. actinomycetemcomitans, T. forsythia, F. nucleatum, P. intermedia* in the PLS samples [33–37]. We also found, however, a higher proportion of *P. oris*, *P. micra* and, *C. Saccharibacteria*. Furthermore, the relative abundance of *A. actinomycetemcomitans* was significantly higher in the PLS patients in our study relative to previous reports [2, 6, 36, 37]. From the above studies one can conclude that *A. actinomycetemcomitans* is the chief oral pathogen responsible for the periodontal disorders of the PLS patients.

As far as we know, a complete microbiological profile of healthy PLS family members has not been presented yet. We compared the oral microbial profile of healthy and diseased PLS family members with those of periodontitis patients as well as adolescents with gingivitis and gingival health, reported earlier [48, 49]. Our results is consistent with the findings of previous investigations, which reported the presence of pathogenic bacterial species in periodontally healthy individuals [73, 74]. Stabholz et al. reported high prevalence of leukotoxic strains of *A. actinomycetemcomitans* in unaffected members of PLS family. Our results are not consistent with these findings, the occurrence of *A. actinomycetemcomitans* was relatively low in healthy family members, but the other important periodontopathogens isolated in higher proportions of affected samples (*T. forsythia, F. nucleatum, P. intermedia, P. oris*, *P. micra* and, *C. Saccharibacteria)* are also notable components of microbiome of healthy family members. This may be due to familial aggregation of periodontal pathogenes, but it seems that *A. actinomycetemcomitans* may be an intrinsic feature of this disease rather than solely inherited from parents, as previously published some authors [44–46, 75].

Several PLS patients have been reported to lack notable immunodeficiency in spite of the missing functional serine protease in PMNs and cytotoxic lymphocytes [76, 77]. The reduced capacity of NET production and higher reactive oxygen species (ROS) formation provide stimulus for the improper recruiting of highly responsive neutrophils in periodontal tissues, acute inflammation and bone loss [78]. Acute inflammatory episodes and recurrent pyogenic infections may occur in PLS patients [50] as we experienced at Patient-B.

Non-surgical periodontal treatment with adjunctive systemic medication is an important therapeutic approach for treatment of active periodontal inflammation [79, 80]. Compared to her elder sister (Patient-B) the periodontal parameters of the younger affected patient (Patient-A) were substantially better as the result of the combined periodontal treatment. Before periodontal therapy Patient-B harboured a less diverse subgingival microbiome. Following the mechanical debridement and low-dose tetracyclin treatment, the composition of subgingival microflora became less diverse, the relative abundance of *A. actinomycetemcomitans* elevated to 62%, *P. oris* increased while *P. intermedia* decreased. Our findings demonstrated a short-term effect of mechanical and antimicrobial therapy. A possible explanation for these apparently unfavourable results could be ineffective oral hygiene, as Patient-B reportedly lost her motivation in personal oral hygiene [80]. Additionally, the systemic antibiotic treatment could induce the emergence of pathogens resistant to antibiotics. Kleinfelder et al. have reported the increased proportion of *A. actinomycetemcomitans* in the microflora of a PLS patient no later than 8 weeks after administration of metronidazole. The repeated medication may result in continuous emergence of resistant microorganisms in the subgingival microbiomes [6]. Jepsen et al. studied the antimicrobial susceptibility of selected periodontopathogens. It is noteworthy, however, that *A. actinomycetemcomitans* is not susceptible to metronidazole [81]. Although *A. actinomycetemcomitans* is susceptible to Doxycyclin the increased relative abundance of *A. actinomycetemcomitans* after the adjunctive administration of this active agent combined with mechanical treatment may suggest that medication with systemic antibiotics at patient with poor oral hygiene result in failed clinical and microbial values, furthermore, likely facilitate selecting of resistant bacterial strains. At any rate, the teeth of Patient-B were saved with moderate attachment loss at the age of 18 years.

A generalized conclusion of this study suggests that the PLS patients apparently differ substantially from subjects of common periodontal inflammatory diseases. An equally important finding indicated that the clinically healthy recessive heterozygote carriers of the CTSC gene mutations show the alterations in the microbiome composition characteristic of mild periodontitis. These observations may trigger the development of specific treatment strategies, e.g., specific probiotic strains/bacteriocins against *A. actinomycetemcomitans*. and to develop more efficient therapeutic protocols for the affected patients. In recent years there have been number of antimicrobial peptids entering clinical trials to treating wound healing in patients with immunodeficiency. The peptide omiganan (MBI226), a12-residue amide derivative of indolicidin (a cathelicidin isolated from bovine neutrophils), and several peptide mimetics are the potential active agent for topical treatment of skin lesion. It is advisable to consider the use of these active substances in controlled-release local delivery antimicrobials in periodontal pockets [82, 83]. Until then, the frequent, meticulous regular mechanical periodontal treatment with deliberated adjunctive systemic administration of antibiotics combined with good individual oral hygiene established with motivation and instruction is an essential condition for successful maintenance therapy. Continuous periodontal maintenance therapy of healthy family members should be considered to avoid cross-infections.

## Data Availability

“Raw amplicon sequences were deposited in NCBI SRA under the bioproject accession number: PRJNA911985.”
